# Application of the patient navigation model with people experiencing homelessness: a scoping review

**DOI:** 10.1093/eurpub/ckac129.422

**Published:** 2022-10-25

**Authors:** I Grabovac, C Carmichael, L Smith, E Aldasoro, A Gil-Salmeron, T Alhambra-Borras, A Donate-Martinez, R Seiler-Ramadas

**Affiliations:** 1 Department of Social and Preventive Medicine, Medical University of Vienna, Vienna, Austria; 2 Centre for Health, Performance and Wellbeing, Anglia Ruskin University, Cambridge, UK; 3 International Foundation for Integrated Care, Oxford, UK; 4 Polibienestar Research Institute, University of Valencia, Valencia, Spain

## Abstract

**Background:**

Barriers in accessing healthcare services are a common issue that contributes to the disproportionately poor health outcomes in people who experience homelessness. A possible way to overcome these barriers and meaningfully engage with this under-served population is through the implementation of the so called patient navigation (PN) models. We conducted a systematic scoping review to gain a better understanding on how PN models are utilized with people experiencing homelessness and other comparable populations and to identify their features, barriers and facilitators to their implementation and their outcomes.

**Methods:**

A systematic scoping review was done based on a predetermined protocol. We conducted a search of Web of Science, PubMed/Medline and Scopus databases on the 15th of June 2021. A narrative analysis of the included studies was conducted.

**Results:**

Our search yielded 1203 hits, and after removing 475 duplicates, we were left with 728 publications of interest. Finally, 21 studies have been included in the review, comprising of nine review articles and 12 individual studies, with most studies stemming from the USA. Results show that PN models are consistently associated with improvements in a wide range of health related outcomes, mostly with timely access to healthcare services. Implementation strategies and measurements used in assessing PN models show heterogeneity between studies. However, a number of consistencies were found including; a longitudinal approach, using of non-clinical navigators who share similarities to the participant groups and who engage as facilitators. Additional considerations for gender and age may further improve the outcomes.

**Conclusions:**

In order to improve on the success of the PN models and their application in removing barriers to healthcare access for people experiencing homelessness, more research is needed that focuses on the feasibility, acceptability and scalability of the approach outside the USA.

**Key messages:**

• People experiencing homelessness experience considerable barriers in accessing healthcare service leading to disproportionately larger burden of disease as well as lower life expectancy.

• Patient navigation models have a great potential in removing barriers and facilitating timely access to healthcare services in people experiencing homelessness.

